# Behavioural and Neural Reliability of a Pavlovian‐to‐Instrumental Transfer Task

**DOI:** 10.1111/adb.70112

**Published:** 2025-12-18

**Authors:** Matthew J. Belanger, Hao Chen, Juliane H. Fröhner, Maria Garbusow, Andreas Heinz, Michael N. Smolka

**Affiliations:** ^1^ Department of Psychiatry and Psychotherapy Technische Universität Dresden Dresden Germany; ^2^ Department of Sociology, Social Policy, and Criminology, Faculty of Social Sciences University of Stirling Stirling UK; ^3^ Department of Psychiatry and Neurosciences Charité – Universitätsmedizin Berlin Berlin Germany; ^4^ Department of Psychology, Clinical Psychology and Psychotherapy MSB Medical School Berlin Berlin Germany

## Abstract

The use of Pavlovian‐to‐instrumental transfer (PIT) in addiction research is on the rise as a means of assessing an individual's susceptibility to interference between Pavlovian and instrumental control over behaviour. However, the reliability of PIT tasks has been rarely assessed.The present study provides an investigation of the reliability of PIT, both on a behavioural and neural level, examining split‐half as well as test–retest reliability. We assessed two different samples: (1) a mixed detoxified alcohol‐dependent sample (with controls) comprising 119 behavioural and 69 fMRI datasets assessed twice within 3–4 weeks and (2) a developmental sample with 117 behavioural and 91 fMRI datasets assessed twice after 3 years. We computed two behavioural parameters of PIT (interference and motivational PIT effects) that were used in our previous studies. The interference PIT effect was assessed as the difference in error rates between the congruent and incongruent trials of the PIT task. The motivational PIT effect was assessed as the linear relationship between the number of button presses influenced by Pavlovian‐conditioned environmental cues. We further assessed the reliability of four predefined fMRI PIT contrasts.On the behavioural level, our results revealed excellent split‐half reliability for both the interference (*r* = 0.92–0.97) and motivational (*r* = 0.94–0.98) aspects of PIT in both clinical and developmental samples. In comparison, test–retest reliability after 3 weeks was lower (clinical sample: ICC = 0.53) and again lower yet still significant despite neurodevelopmental brain maturation after 3 years (developmental sample: ICC = 0.26–0.29). In the fMRI analysis, regions of interest showed acceptable ICCs in the incongruent and congruent contrasts (split‐half: 0.59–0.80; test–retest: 0.13–0.51). Global overlap assessments using Jaccard coefficients revealed individual‐level variability in neural responses (split‐half: 47%–51% overlap; test–retest: 29%–35% depending on the sample and contrast). All fMRI reliability coefficients for the motivational PIT effect were below 0.17.Overall, behavioural PIT reliability was good, especially from the split‐half perspective. For neuroimaging, the incongruent contrast seems best suited for predicting individual outcomes, while the neural motivational PIT effect seems to represent more changeable current states.

## Graphical abstract

The current study tested the reliability of behavioural and neural measures of Pavlovian‐to‐Instrumental Transfer after three weeks and three years. We found high behavioural split‐half reliability but lower test‐retest stability. Neural reliability was acceptable for the incongruent and congruent contrasts. The incongruent contrast seems best suited for predicting individual outcomes, while the neural motivational and interference PIT effects seem to represent more changeable states.

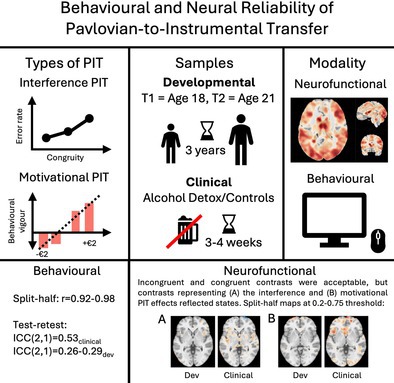

## 1 Introduction

The consumption of drugs can lead to the development of automatic and implicit responses to environmental cues through associative learning processes. In the context of addiction, this can involve the association of drug‐related cues with the pleasurable effects of drug use, leading to drug craving and drug‐seeking behaviour [1, 2]. Empirical studies indicate that drug seeking often arises from automatic, nonconscious processes triggered directly by environmental or drug‐related cues, which can drive behaviour even in the absence of conscious craving [3, 4, 5]. The interaction of Pavlovian and instrumental control is particularly relevant in this context, as the automatic response tendencies elicited by Pavlovian cues can bias goal‐directed instrumental behaviour (i.e., do not enter your favourite bar) in maladaptive directions, leading to drug‐seeking and taking [6]. This dysregulation of goal‐directed control is a key mechanism that promotes the development of substance use disorders [7]. This proves harmful to an individual as the initiation of their approach behaviour toward drugs is frequently not mediated by a conscious process [8] and hinders an individual's ability to regain control.

Pavlovian‐to‐instrumental transfer (PIT) tasks are well suited to serve as an index to quantify how susceptible an individual's goal‐directed instrumental behaviour is to the influence of Pavlovian cues. In contrast to traditional measures of cue reactivity, such as heart rate, questionnaires and electrophysiological responses, PIT allows researchers to directly observe changes in instrumental behaviour by those cues. This enables a deeper understanding of the impact of the Pavlovian influence on behaviour, which can ultimately aid in the development of interventions that disrupt these effects, such as just‐in‐time adaptive interventions [9]. While tasks like PIT offer insights into the neurocognitive mechanisms of addiction, their utility for clinical translation depends heavily on their psychometric properties. In particular, the reliability of task‐based fMRI measures determines whether they can meaningfully contribute to outcome prediction or tracking individual changes. Such tasks may only provide a mechanistic understanding without practical clinical application if they lack robust psychometric properties.

Our research group has utilized PIT to investigate several research questions regarding alcohol use and dependence over the past decade. Beginning in 2014, we first developed and tested a modified PIT paradigm from Huys et al. [10] using both control individuals and those with diagnosed alcohol dependence (according to DSM‐IV‐R criteria), finding that the latter group was more susceptible to the PIT effect, particularly in reaction to aversive cues [11]. In 2016, we investigated detoxified alcohol‐dependent patients and discovered that the pronounced PIT effect they exhibited was predictive of their alcohol consumption and their short‐term relapse likelihood [12]. Further insights with respect to PIT resulted from a longitudinal study we performed using a developmental sample. By age 18, we demonstrated that high‐risk drinkers were particularly susceptible to Pavlovian conditioned cues, with associated brain regions such as the amygdala [13], ventral striatum (VS), as well as the lateral and dorsomedial prefrontal cortices (lPFC and dmPFC) potentially mediating this behaviour [14]. More recently, in 2023, our group further elucidated the developmental trajectory of alcohol consumption behaviours over 6 years, establishing that susceptibility to Pavlovian interference during young adulthood could predict hazardous drinking behaviours in the years that follow [15]. However, contrasting findings suggest that goal‐directed control and PIT remain intact in abstinent individuals with alcohol use disorder, with no significant behavioural or neurobiological differences from healthy controls [16].

In our single‐lever task, participants performed an instrumental action (repeated button presses) to obtain rewards. The button‐press behaviour was further influenced by the presence of Pavlovian‐conditioned cues associated with the gain and loss of money. In the past, we have used two approaches to assess the behavioural PIT effect: the interference and the motivational PIT effects. The interference effect manifests during task trials where instrumental and Pavlovian cues possess incongruent valences, typically resulting in higher error rates compared to trials with congruent valences. The phenomenon of conflicting values emerges when an appetitive instrumental cue is paired with a negative Pavlovian cue, or vice versa. In our paradigm, an appetitive instrumental cue is one that elicits approach behaviour to gain reward and avoidance to avert loss. The motivational effect was measured as one regression coefficient between button presses and all Pavlovian cue values. More specific details can be found in the Methods section. It is important to note that while both the motivation and interference approaches have been shown to be highly correlated on a behavioural level, they are reflected by activity in distinct underlying neural networks associated with reward‐related motivational functioning on the one hand and cognitive control on the other [14]. This potential dissociation in neural underpinnings motivates our investigation of both approaches in the present study.

In psychology, reliability is defined as the ratio of true score variance to the total variance [17, 18]. It refers to the degree to which measures are free from error such that they would produce consistent results if taken again. Test–retest reliability involves administering the same measure to a group of individuals at two different points in time and comparing the results to assess the stability of the measure over time [19]. The basic assumption is that the true score does not change over time, but this assumption is violated for many variables that are not perfect traits or for variables that exhibit slow change over time as typical in development or aging processes. For variables that fluctuate (e.g., states), other approaches to assessing reliability are better suited. For example, split‐half reliability involves dividing a single measure into two halves and comparing the results of each half to assess the internal consistency.

To assess cognitive processes and subsequently draw conclusions in a manner that is appropriate for the research question, especially concerning individual differences, reliable measures are requisite. Historically, reliability metrics of behavioural assessments of cognitive functions (i.e., Stroop, go‐no‐go or working memory tasks) were not systematically assessed and are rarely reported within studies, which made it difficult to ascertain whether certain results represented real effects or were the result of a sampling error or measurement bias. Presently, the inclusion of reliability metrics is becoming more common, largely because of Open Science initiatives [20]. Despite the century‐long existence of modern reliability research [21, 22], there still lacks a standardized, systematic approach to assess the reliability of cognitive‐behavioural measures and imaging data from cognitive neuroscience. Parsons et al. [23] suggest the use of a permutation‐based split‐half estimation procedure and intraclass correlation coefficients (ICC) to assess internal consistency and test–retest reliability.

This shortcoming extends beyond behavioural data; task‐based fMRI studies also have a great deal of variability in their results and use partly different reliability metrics. Quantifying the reliability of fMRI data is a challenge in general, as results are easily influenced by factors such as experimental design, measurement interval, contrast type, scan length or even resting condition [24, 25, 26, 27]. One can find within existing literature a mixture of foci with respect to reliability analyses. Some focused on group‐level analyses [28, 29], while others considered within‐subject variance [30, 31]. Recent meta‐analyses conclude that fMRI reliability is generally poor [32, 33]. To follow guidelines previously proposed by our group, the analysis presented herein will provide a comprehensive overview of behavioural and fMRI PIT reliability, focusing on individual‐ and group‐level effects across and between sessions [34]. While our group previously explored the reliability of behavioural PIT effects in pilot samples showing moderate to good reliability [11, 35], we now aim to investigate reliability in larger samples and additionally on a neural level.

## 2 Materials and Methods

We analysed all PIT data, which originated from a larger longitudinal study funded by the German Research Foundation beginning in 2012 and ending in 2020 (Project Number: 186318919). This project investigated the role of learning in the development of alcohol dependence, exploring how Pavlovian values and habits influence choices in both at‐risk young adults and alcohol‐dependent patients. Our “developmental sample” therefore included a randomly selected group of 18‐year‐old males from Dresden and Berlin, Germany (for more details, see [14]) measured twice with the PIT task after 3 years (mean: 3.13 years). Our ‘clinical’ sample included PIT data from controls in addition to individuals (of varying ages and genders) diagnosed with alcohol dependence measured twice with the PIT task within approximately 3 weeks (mean: 24 days, or 3.43 weeks). For the developmental sample, a total of 91 fMRI datasets and 117 behavioural datasets were included in the analysis. For the clinical sample, there were 119 behavioural and 69 fMRI datasets. For an overview of relevant characteristics of the samples, see Table 1.

**TABLE 1:** Sample characteristics of the developmental and clinical samples.

Sample	Age^a^	Gender	Binge drinking score^b^, mean (SD)	AUDIT^c^, mean (SD)	Education^d^, mean (SD)	Employment^e^, mean (SD)	Socioeconomic status^f^	DSM‐IV alcohol dependence symptoms^g^, mean (SD)
Developmental	18	100% Male	54 (38.78)	6.22 (4.57)	1.27 (1.19)	1.27 (0.70)	2.09 (0.65)	—
	21	100% Male	36 (35.18)	5.80 (3.65)	2.80 (1.13)	1.74 (1.00)	1.98 (0.66)	—
Clinical	47.56	82.54% Male	171 (161.49)	19.67 (12.03)	3.13 (0.86)	1.87 (1.36)	1.59 (0.65)	4.19 (1.21)

^a^ Mean age is given for the clinical sample. In the developmental sample, all participants were assessed at ages 18 and 21.

^b^ Binge drinking score is measured as the number of grams of alcohol consumed per drinking occasion over the past year.

^c^ The Alcohol Use Disorders Identification Test (AUDIT) score assesses the severity of alcohol use, with total scores ranging from 0 to 40, where higher scores indicate greater levels of hazardous or harmful drinking behaviour. AUDIT was assessed at age 18.5.

^d^ 1—none, 2—currently in vocational training, 3—currently studying at university, 4—currently pursuing a doctorate/habilitation, 5—completed vocational training, 6—vocational training discontinued, 7—technical school (e.g., master craftsman), 8—bachelor's degree discontinued, 9—bachelor's degree, 10—master's degree discontinued, 11—master's degree, 12—diploma/magister discontinued, 13—diploma/magister degree, 14—doctorate/habilitation discontinued, 15—doctorate/habilitation.

^e^ 0—Unemployed, 1—student, 3—employed.

^f^ 0—Lower, 1—lower, 2—middle, 3—upper middle, 4—upper class.

^g^ Scores range from 0 to 7, with higher values indicating a greater number of dependence symptoms.

### 2.1 Task Description

**FIGURE 1:**
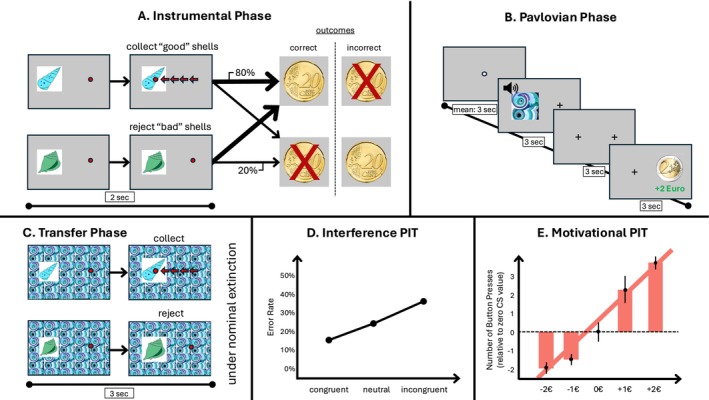
(A) Instrumental phase. Participants learned to collect good shells and avoid bad shells using the mouse button, which moved a dot toward the shell when repeatedly pressed. Training comprised 60 trials and ended when participants reached 80% correct choices over the last 16 consecutive trials or after a maximum of 120 trials. (B) Pavlovian phase. Participants passively viewed compound conditioned stimuli consisting of fractal images and tones that predicted positive, negative or neutral outcomes. This phase included 80 trials, with 16 trials for each stimulus type. (C) PIT phase. Participants performed the instrumental task again while the conditioned stimuli were displayed in the background. Each trial lasted 3 s, with conditioned stimuli presented 0.6 s before the instrumental shells, leaving a 2.4‐s response window. This phase comprised 90 trials and was conducted under nominal extinction. All participants completed the same task version with identical timings. The task was administered at the neuroimaging centres in Dresden and Berlin using similar computer hardware, and instructions were presented on screen to ensure each participant received the same guidance. The PIT task formed part of a longer testing session, and sessions were typically scheduled in the late morning or early afternoon, but the session times were not standardized. (D–E) We also demonstrate the hypothesised outcome of both the interference and motivational analysis approaches of PIT.

In this task, participants learned the intrinsic qualities of shells through trial and error by collecting ‘good’ or rejecting ‘bad’ shells using a standard computer mouse. They received probabilistic visual feedback indicating whether they received a monetary gain or loss. During the Pavlovian learning phase, the participants were presented with compound audiovisual stimuli, which consisted of a fractal‐like, distorted image and a pure tone. Each fractal and tone pairing was randomly associated with either a positive (+1€ or +2€), neutral (0€) or negative outcome (−1€ or −2€). During the PIT phase, the participants were asked to once again collect or reject the shells, but now with images tiled in the background. Please refer to [11, 12] or Chen et al. [15] for a more detailed description of the task (Figure 1).

### 2.2 Behavioural Data

We analysed the reliability of two behavioural indicators of the PIT effect: the error rate difference (incongruent—congruent) and the linear regression coefficient between button presses and the monetary value assigned to Pavlovian cues. The error rate difference reflects the interference PIT effect, where Pavlovian cues influence instrumental behaviours and potentially lead to maladaptive choices. The regression coefficient represents the motivational PIT effect, which quantifies changes in response vigour (i.e., the number of button presses) based on cue association. To assess the motivational PIT effect, we used a generalized linear mixed‐effects model with a Poisson distribution for count data. This model is featured in Chen et al. [36] and Ebrahimi et al. [37]. The model examined the effect of the monetary value assigned to Pavlovian cues (*PavlovianValue*) and whether participants approached or avoided the instrumental stimulus *(InstrumentalResponse*_*GoNogo*) on the number of button presses (*ButtonPress*). Random slopes and intercepts were included for each participant (*Participant_ID*), and random intercepts were specified for both the Pavlovian and instrumental cues (*PavlovianCue* and *InstrumentCue*) to account for cue‐level variability. The model was fit using the bobyqa optimizer and is specified as follows:

$$\begin{array}{c} \text{glmer}(\text{ButtonPress}\sim \text{PavlovianValue}+\text{InstrumentalRespons}e_{\text{GoNogo}}+\\ \left(\text{PavlovianValue}+\text{InstrumentalRespons}e_{\text{GoNogo}}|\text{Participant}\_\mathrm{ID}\right)+\\ \left(1|\text{PavlovianCue}\right)+\left(1|\text{InstrumentalCue}\right) \end{array}$$

### 2.3 Behavioural Analysis: Split‐Half Reliability and Test–Retest Stability

To calculate the split‐half reliability coefficients for the behavioural data, the data from each time point were randomly split and the Pearson product–moment correlation was calculated between the two splits. In accordance with recent recommendations [23], the reported split‐half reliability coefficients were based on 5000 permutations. A Spearman–Brown correction was applied to the coefficients to account for underestimation due to the reduced number of items. Fisher's z‐transformation was applied to the corrected coefficients before taking the mean of the 5000 permutations, after which the values were transformed back into their original scale.

Longitudinal studies often require significant time between measurements, which is essential for developmental studies. In this study, the test–retest (longitudinal) stability of both PIT measures is represented by the agreement ICC. ICC estimates and their 95% confidence intervals were calculated in R Studio using the ‘irr’ package [38] based on a single‐measurement, absolute‐agreement, two‐way mixed‐effects model. Given the long period between both measures, the absolute agreement assumption may underestimate the reliability. Regardless, we have chosen to use the more conservative approach but also report the Pearson correlation coefficients between time points for each variable, as these are unaffected by group‐level changes. Further, in the context of the 3‐year retest analyses, we refer to these results as reflecting ‘stability,’ as these longitudinal estimates capture more than measurement error alone. It is clear that there are many sources of variance besides measurement error over 3 years (i.e., trait changes, state changes or behavioural adaptations). For this reason, the 3‐year findings do not represent a clean estimate of measurement error and should not be discussed as such. Instead, they provide an indication of longer‐term stability.

### 2.4 fMRI Data

#### 2.4.1 Image Acquisition and Preprocessing

Functional imaging was performed with a Siemens 3‐Tesla MRI scanner (Magnetom Trio, Siemens, Erlangen, Germany) with an echo planar imaging (EPI) sequence (repetition time [TR]: 2410 ms; echo time [TE]: 25 ms; flip angle: 80°; number of slices: 42; slice thickness: 2 mm [1‐mm gap]; field of view [FoV]: 192 x 192 mm^2^; voxel size: 3 x 3 x 2 mm^3^; 480 volumes). The preprocessing of the fMRI data was performed with Nipype [39]. The EPI images were slice‐time corrected, realigned, and coregistered to each individual's segmented and normalized structural image. They were then smoothed with a Gaussian kernel (8‐mm full width at half maximum). The fMRI data collected during the PIT phase was analyzed with the general linear model (GLM) in SPM12 (Wellcome Trust Centre for Neuroimaging, London, UK).

#### 2.4.2 First Level Statistics

##### 2.4.2.1 Interference PIT Model

The interference PIT model was constructed in the same way as that described by Chen *et al*. [14]. The model comprised 10 onset regressors of interest: five distinct Pavlovian conditioned stimulus (CS) values (€‐2, −1, 0, +1 and +2) combined with two instrumental conditions (collect and reject). Despite the inclusion of regressors for the alcohol/water trials, these were deemed irrelevant to the present analysis. Additionally, a regressor for the onset of each registered button press was incorporated as a stick function, along with the six motion regressors. The interference PIT effect in this model thus can be defined as incongruent versus congruent conditions. Incongruent trials represent the conditions where participants need to collect a good shell during the presentation of negatively valenced Pavlovian cues (€‐1 or −2) or reject a bad shell during positively valenced cue presentation.

##### 2.4.2.2 Motivational PIT Model

The motivational PIT first‐level model was constructed in the same way as in Chen et al. [15]. The model includes one onset regressor for all monetary PIT trials, modulated with three parametric regressors: the interaction of Pavlovian CS value and the log‐transformed number of button presses (representing the motivational PIT effect), Pavlovian CS values alone and the log‐transformed number of button presses alone. The model also includes the alcohol/water PIT trials, which are not relevant to the current analysis. Additionally, the model incorporates a regressor for each registered button press, modelled as a stick function, alongside six motion regressors to account for movement. The parametric modulators were not orthogonalized with respect to the main regressors or to each other to avoid order dependency and to preserve clear interpretability of their effects. For a more detailed explanation of this decision, please see the Supplementary Material Section S1.

### 2.5 fMRI Reliability

#### 2.5.1 fMRI Reliability Toolbox and Contrasts of Interest

The split‐half and test–retest reliability were assessed using a MATLAB/SPM12‐based, open‐access toolbox called *fmreli* [34] (MATLAB: Mathworks Inc., Sherborn, Massachusetts, USA). This toolbox is available via GitHub (https://github.com/nkroemer/reliability) and is operated via a MATLAB‐based graphical user interface.

In the interference PIT model, the main contrasts of interest that we investigated for reliability were the congruent, incongruent and incongruent versus congruent contrasts. The congruent and incongruent contrasts correspond to brain regions eliciting responses to harmony and conflict between Pavlovian cues and instrumental behaviours, respectively. The Incongruent versus songruent contrast further delineates brain responses to conflict by using the congruent condition as the baseline (i.e., this contrast reflects brain responses specifically related to conflict processing by subtracting the congruent condition signal from the incongruent signal). When faced with incongruent instrumental and Pavlovian cues, areas associated with decision‐making and conflict resolution, such as the dorsomedial prefrontal cortex (dmPFC), are also of interest [40].

In the motivational PIT model, the main contrasts of interest include the monetary trial onset, which provides insights into how participants process monetary cues. Additionally, we investigated the contrast of the parametric modulator of the interaction between Pavlovian CS values and the transformed number of button presses, representing the motivational PIT effect. We also examined the other two parametric modulators (CS values and button presses), which elucidate the processing of Pavlovian values and motor associations, respectively. Here, brain regions associated with reward processing and motivation, such as the VS, may be of interest [41]. Therefore, we employ both models to assess the reliability of both aspects of PIT.

#### 2.5.2 fMRI Reliability Measures

The analysis of the fMRI data employs a method that both cross‐sectionally and longitudinally evaluates reliability based on the methodology suggested by Fröhner et al. [34]. To assess cross‐sectional reliability, this method integrates a random split‐half approach. Therefore, the regressors of interest are randomly split into two halves before re‐estimating first‐level statistics. Although the application of split‐half methodology to fMRI analysis is relatively novel, its utility is evidenced by recent studies [42, 43, 44, 45, 46].

First, we assessed local reliability by computing (local) absolute‐agreement ICCs for four predefined regions of interest (ROIs) based on our previous work [14, 15]. To do this, we extracted unweighted average beta values and computed their mean for the amygdala, the VS and the lateral and dorsomedial prefrontal cortices (lPFC and dmPFC). We did this for each session's split and original data. For completeness, voxel‐wise reliability results (split‐half and test–retest) are reported in the supplementary information.

Following the regional reliability assessment, global reliability was examined via an overlap analysis using the Jaccard coefficient [47, 48]. This analysis represents the reliability at the whole‐brain level across all contrast conditions by determining the extent of voxel overlap between split or two scanning sessions. The Jaccard coefficient can be readily interpreted as a percentage of overlap and is calculated by dividing the number of overlapping voxels by the total number of significant voxels shared between the two sessions. The Jaccard coefficient is an established measure of overlap and is expressed on a scale from 0 to 1, with 1 representing perfect overlap. In the current study, we defined a threshold of *p* < 0.01, but for comparison purposes, we further report an overview and interpretation of the results with more liberal and conservative thresholds of *p* < 0.05 and *p* < 0.001.

## 3 Results

### 3.1 Behavioural Data

#### 3.1.1 Split‐Half Reliability

For both the interference PIT effect and the motivational PIT effect, split‐half reliability was excellent across both samples and time points. Values ranged from *r* = 0.90 to *r* = 0.97. For a summary, refer to Table 2. A summary of the distribution of values obtained from the permutation tests is given in the Supplementary Material (Table S2 and Figure S3).

**TABLE 2:** Summary of the split‐half and test–retest behavioural reliability analysis.

Reliability/stability	Interference PIT effect		Motivational PIT effect	
	Clinical	Dev	Clinical	Dev
Split‐half T1	0.95	0.92	0.97	0.94
Split‐half T2	0.97	0.95	0.98	0.96
Test–retest	0.53	0.29	0.53	0.26

*Note:* Split‐half reliability was computed via Pearson's correlation and Spearman‐Brown corrected. Test–retest reliabilities are intraclass coefficients (ICC). Classification of ICCs according to Koo and Li [49] poor ≤ 0.5, 0.5 ≤ 0.75 moderate, 0.75 ≤ 0.9 good and excellent ≥ 0.9. We colour‐code these results according to Cicchetti and Sparrow's [50] more liberal guidelines, also applied to split‐half reliabilities: 0.1 < poor < 0.4, 0.4 ≤ fair < 0.6, 0.6 ≤ good < 0.75 and excellent ≥ 0.75. Clinical, clinical sample; dev, developmental sample.

#### 3.1.2 Test–Retest Reliability

Koo and Li [49] recommend interpreting ICC values below 0.50 as poor, between 0.50 and 0.75 as moderate, between 0.75 and 0.90 as good and above 0.90 as excellent. However, these thresholds were not designed for, nor empirically validated against, the distinct statistical properties and noise characteristics of BOLD fMRI signals. Applying these thresholds to task‐fMRI data risks mischaracterizing the reliability profile. Until field‐specific criteria are developed, we contextualize our findings with respect to the large‐scale meta‐analysis by Elliott et al. [32]. Compared to the split‐half coefficients, test–retest reliability of the interference PIT effect was lower after 3 weeks in the clinical sample (ICC = 0.53) and even lower after 3 years in the developmental sample (ICC = 0.29). Similarly, the motivational PIT effect showed ICCs of 0.53 for the clinical sample and 0.26 for the developmental sample. To assess potential change over time, paired‐sample *t* tests were conducted. The interference PIT effect did not significantly change in the clinical sample (*t*(118) = 1.07, *p* = 0.29) or the developmental sample (*t*(116) = −0.56, *p* = 0.58). Similarly, the motivational PIT effect did not significantly change in the clinical sample (*t*(118) = 1.97, *p* = 0.05) or in the developmental sample (*t*(116) = 0.46, *p* = 0.65), although the *p* value of the former was close to the conventional threshold for significance.

### 3.2 fMRI Reliability

#### 3.2.1 Local Reliability (ICC)

At the local (voxel) level, we summarized the split‐half, and longitudinal voxel‐wise ICCs for both developmental and clinical samples. The results of this analysis are stored in an online repository, and the unthresholded ICC maps can be displayed interactively (https://neurovault.org/collections/KJYQNYJO/). In line with the ROI‐based analyses and previous findings, cortical regions exhibited higher reliability than subcortical regions. Split‐half reliability indices were generally higher than test–retest indices, and onset contrasts showed greater reliability than difference or parametric contrasts. The repository enables readers to examine reliability estimates for specific brain regions of interest and to assess the robustness of the present findings.

#### 3.2.2 Regional Reliability

The ICC results for the four ROIs are shown in Table 3. Both the incongruent and congruent contrasts exhibited good split‐half reliability across all four ROIs for both samples, with ICCs ≥ 0.47 (with many above 0.60). As a reference, most of the monetary onset contrasts also showed fair reliability. However, the reliability of the remaining contrasts did not meet acceptable standards. The split‐half reliability appears to be higher for the congruent and incongruent contrasts than for the other contrasts.

**TABLE 3:** Regional and global reliability/stability of fMRI data.

		Incongruent		Congruent		Incongruent vs. congruent		Monetary trial onset		Motivation PIT effect (parametric)	
		**Clinical**	**Dev**	**Clinical**	**Dev**	**Clinical**	**Dev**	**Clinical**	**Dev**	**Clinical**	**Dev**
**Split‐half T1**	**Amy**	0.666	0.548	0.586	0.667	0.091	−0.082	0.122	0.373	−0.007	−0.017
	**VS**	0.639	0.468	0.671	0.624	0.086	−0.002	0.295	0.443	0.071	0.027
	**lPFC**	0.603	0.587	0.709	0.723	0.075	0.092	0.461	0.475	−0.088	0.107
	**dmPFC**	0.484	0.545	0.591	0.688	0.019	0.119	0.526	0.474	−0.048	0.055
	***Global p < 0.01***	0.485	0.468	0.482	0.486	0.002	0.002	0.319	0.340	0.005	0.002
**Split‐half T2**	**Amy**	0.706	0.550	0.640	0.620	−0.004	0.112	0.071	0.461	0.076	−0.067
	**VS**	0.701	0.582	0.607	0.587	−0.007	0.001	−0.060	0.333	0.034	−0.065
	**lPFC**	0.798	0.624	0.676	0.711	0.223	0.010	0.415	0.397	0.001	−0.009
	**dmPFC**	0.729	0.545	0.639	0.669	0.097	−0.008	0.492	0.328	−0.032	−0.157
	***Global p < 0.01***	0.509	0.491	0.474	0.489	0.004	0.003	0.285	0.341	0.004	0.003
**Test–retest**	**Amy**	0.134	0.282	0.164	0.267	0.009	−0.055	0.073	0.072	0.055	−0.054
	**VS**	0.285	0.330	0.302	0.253	−0.051	−0.136	0.198	0.116	0.170	−0.035
	**lPFC**	0.421	0.419	0.386	0.511	−0.077	−0.119	0.550	0.272	−0.029	0.012
	**dmPFC**	0.401	0.360	0.368	0.381	−0.040	−0.070	0.577	0.192	−0.116	0.032
	***Global p < 0.01***	0.352	0.349	0.289	0.336	0.001	0.003	0.271	0.284	0.003	0.002

*Note:* Global coefficients represent the Jaccard coefficient, while all other values indicate the mean ICC for each region. Classification of ICCs according to Koo and Li [49] poor ≤ 0.5, 0.5 ≤ 0.75 moderate, 0.75 ≤ 0.9 good, excellent ≥ 0.9. We colour‐code these results according to Cicchetti and Sparrow's [50] more liberal guidelines [50] 0.1 ≤ poor < 0.4, 0.4 ≤ fair < 0.6, 0.6 ≤ good < 0.75, excellent ≥ 0.75. For the sake of readability, an extended version of this table appears in the Supplementary Material (Table S4A,B) including the confidence intervals and global coefficients at *p* < 0.05 and *p* < 0.001.

Abbreviations: Amy, amygdala; Clinical, clinical sample; Dev, developmental sample; dmPFC, dorsomedial prefrontal cortex; lPFC, lateral prefrontal cortex; VS, ventral striatum.

**FIGURE 2:**
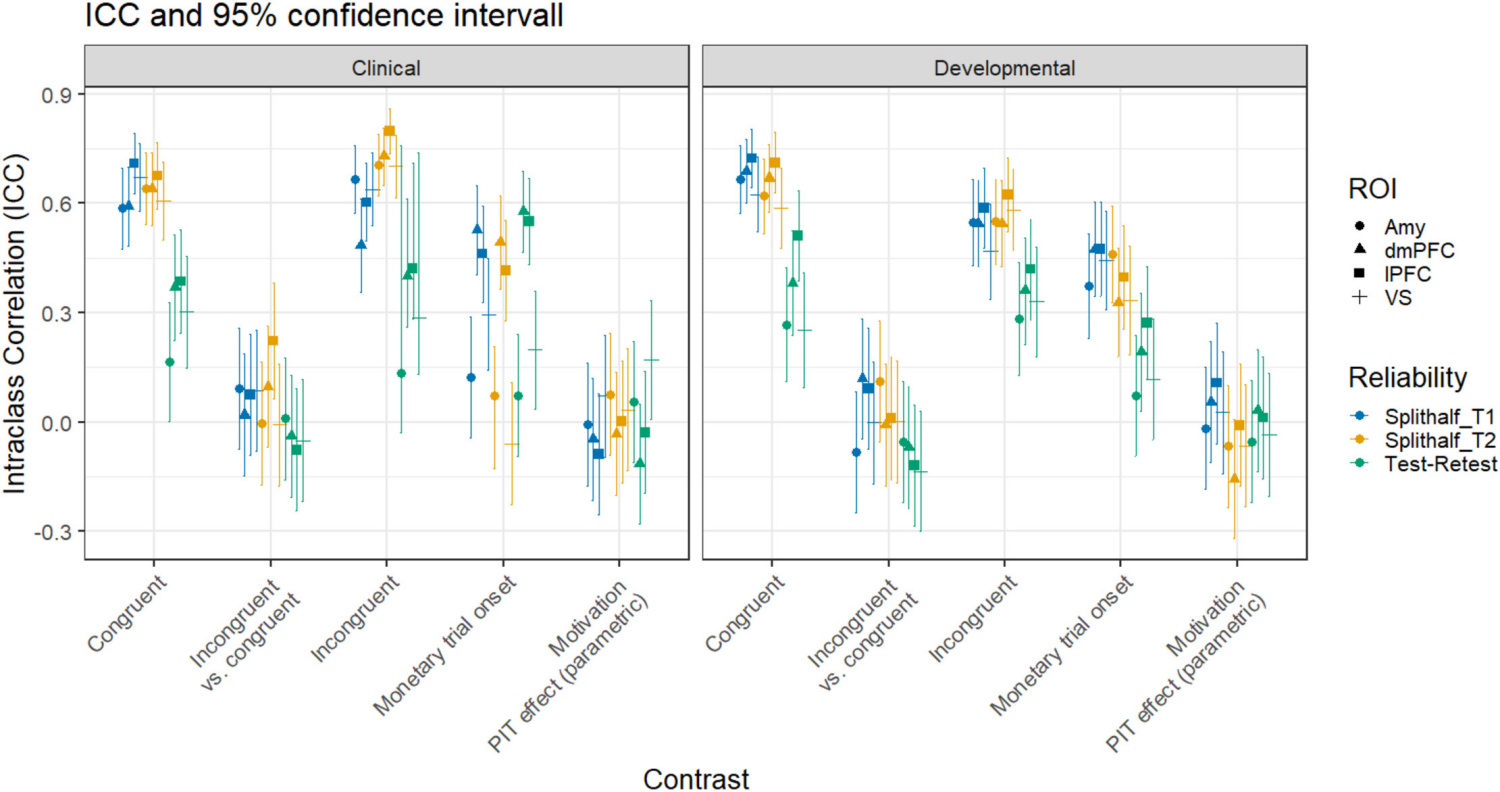
Regional and global reliability/stability estimates for each region of interest. Confidence intervals are represented with confidence bars.

In terms of longitudinal data, the incongruent contrast exhibited ICC values mostly above 0.3 in the VS, lPFC and dmPFC in both samples. As our ROIs include subcortical regions, we interpret these results according to region‐specific norms. An ICC greater than 0.3, though classified as poor by conventional clinical standards, reflects a level of stability that is typical, and in some cases comparatively robust, for subcortical task‐fMRI measures. It is from this perspective that we frame its suitability for longitudinal comparison in the present study. Importantly, these neural responses have previously been associated with the interference PIT effect [14]. Similarly, the congruent contrast in the aforementioned regions yielded ICC values that were also mostly exceeding 0.3. Other contrasts in these ROIs did not meet this stability threshold. The onset of monetary trials also showed fair ICCs in the lPFC and dmPFC for the clinical sample, but not for the developmental sample where the interval between assessments was much longer (3 years vs. 3 weeks). Conversely, the incongruent versus congruent contrasts and the motivational PIT effect contrasts scarcely met the set threshold (Figure 2).

#### 3.2.3 Global Reliability (Overlap)

We computed the split‐half and test–retest Jaccard coefficients at the individual level, including all voxels below thresholds of *p*_uncorrected_ < 0.05, *p*_uncorrected_ < 0.01 and *p*_uncorrected_ < 0.001. Refer to Table 3 for the full summary of the global results. For the split‐half overlap, the Jaccard coefficients revealed good performance of the congruent and incongruent contrasts at the individual level, with Jaccard coefficients ranging from 47% to 51%, depending on the sample and contrast. Here, the congruent and incongruent contrasts also outperformed the monetary trial onset. As with the previous analysis, the incongruent versus congruent contrast, the motivational PIT effect contrast, and the value parametric regressor exhibited minimal overlap.

For the test–retest reliability, the Jaccard coefficients demonstrated that approximately 34%–35% of the significant voxels for the incongruent and congruent contrasts exhibited overlap at the individual level in the developmental sample. In the clinical sample, these values were approximately 29%–35%, indicating a similar result. Once more, the monetary trial onset contrast was used as the reference point, exhibiting an overlap of 27%–28% in both samples. However, the incongruent versus congruent contrast and the motivational PIT effect contrast demonstrated almost no longitudinal overlap, resulting in Jaccard coefficients of approximately 0.

 For an overview of different Jaccard thresholds, please refer to Table S4A in the Supplementary Material.

## 4 Discussion

This study investigated the reliability of PIT in both behavioural and fMRI data across two different groups: a developmental sample and a clinical sample comprising recently detoxified alcohol‐dependent patients and controls. We computed two behavioural measures of PIT and their neural correlates (i.e., interference and motivational). Behaviourally, we found excellent cross‐sectional split‐half reliability and as expected lower test–retest reliability over time, particularly in the developmental group with a retest interval of 3 years. fMRI data showed poor to excellent split‐half reliability in a priori regions of interest and lower reliability at the global level. The test–retest reliability coefficients for fMRI contrasts were again found to be lower than the corresponding split‐half reliability coefficients.

### 4.1 Behavioural Reliability

In the behavioural analyses, both the interference and motivational PIT parameters demonstrated excellent split‐half reliability in both the clinical and developmental samples. Our study also assessed the test–retest reliability of PIT after 3 weeks and 3 years to examine longitudinal differences. The test–retest reliability observed in the sample of detoxified alcohol‐dependent patients and controls over 3 weeks was moderate (Interference: ICC(2,1) = 0.53; Motivational: ICC(2,1) = 0.54). We interpret this result in consideration that substantial changes, including increased glucose metabolism [51], recovery of brain volume [52, 53, 54] and improvements in neuropsychological functioning [55] are typically observed after alcohol detoxification. Thus, the assumption of unchanged true values, on which the estimation of test–retest reliability is based, is probably not fulfilled and results in underestimation of reliability.

In the developmental sample, the test–retest reliability over 3 years was classified as poor according to standard guidelines, but contextually, we find this result to be remarkable for a developmental sample over a substantial period (Interference: ICC(2,1) = 0.29, *p* < 0.001; motivational: ICC(2,1) = 0.26, *p* = 0.002). While a paired *t* test confirmed no differences between both time points, the observed stability is noteworthy considering the developmental changes that are typical in the age group of our sample and the substantial difference in time between measurements. This suggests that PIT may have some degree of stability despite the cognitive and behavioural changes that occur during young adulthood.

We now compare the test–retest reliability of the PIT effect in our study to that of other psychological tasks assessed over short‐ and long‐term intervals. In general, many well‐established cognitive tasks fail to reliably capture individual differences due to low between‐subject variability. Hedge et al. [56] examined the test–retest reliability over 3 weeks of the Eriksen Flanker, Stroop, stop‐signal, go/no‐go, Posner cueing, Navon and Spatial‐Numerical Association of Response Code tasks. The test–retest reliabilities ranged from moderate for the Eriksen Flanker (ICC = 0.40–0.72) and Stroop tasks (ICC = 0.44–0.66) to lower for the stop‐signal (ICC = 0.36–0.49) and Spatial‐Numerical Association of Response Code tasks (ICC = 0.03–0.22). The go/no‐go task showed relatively higher reliability (ICC = 0.76), while the Navon task exhibited considerable variability depending on the measured construct (ICC = 0–0.82) [56]. Further, Smithies et al. [57] found that the Category Switch Task exhibited test–retest reliability for response time measures comparable to other task‐switching paradigms, while accuracy‐based reliability remained lower, with moderate to high reliability observed across same‐day, 1‐day and 1‐week intervals. Similar to the observations of Beglinger et al. [58] and Duff et al. [59], practice effects were more pronounced over shorter intervals (i.e., particularly between same‐day and one‐day assessments), but tended to stabilize by the 1‐week retest. The lower test–retest reliability of the PIT effect we observed in our study (3 weeks vs. 3 years) aligns with the results described by Smithies et al. [57], in that other well‐established cognitive tasks may show limited reliability when assessed over extended periods. Concerning longer test–retest delays, a study examining computerized neurocognitive testing in high school athletes over a 1‐year interval found low to marginal reliability, with ICCs ranging from 0.401 to 0.672 [60]. Additionally, a meta‐analysis of the Serial Reaction Time Task found that despite producing robust procedural learning effects at the group level, its test–retest reliability was consistently low (*r* < 0.40). However, split‐half reliability within a session was higher (*r* = 0.66) [61]. Our results are consistent with these studies.

Garbusow et al. [35] assessed the test–retest reliability of their PIT task using 20 healthy volunteers who completed two parallel versions of the task within 2 days. Their results mirrored ours and demonstrated moderate test–retest PIT reliability (ICC2,1 = 0.54), indicating that the task has satisfactory psychometric properties for clinical studies and interventions.

During detoxification, the brain undergoes substantial changes. These changes would reduce the test–retest estimates due to changes in true values. Considering the excellent split‐half reliability, our test–retest results suggest that the observed differences are, therefore, likely to reflect genuine changes in the underlying true scores rather than random fluctuations, provided we assume no correlated error variance. The contrast between the 3‐week and 3‐year test–retest results (of both the interference and motivational PIT parameters) may also suggest that PIT could have properties of a dynamic state rather than a static trait. The PIT task in our study might, therefore, be capturing participants' susceptibility to PIT cues at the time of testing, which could fluctuate depending on various factors, such as stressful life challenges. A recent study examining the test–retest reliability of response inhibition measures (using the stop signal task) found low temporal stability across three independent studies with a test–retest interval ranging from 1 to 2 years. The authors concluded that inhibition measures were more influenced by state‐like and situational factors than by stable, dispositional traits, with higher stability observed for behavioural and noninhibitory processing measures [62]. In our study's context, conceptualizing PIT as a state‐dependent measure, changes between measurements could be reflected by the observed changes. As an example, the Beck Depression Inventory (BDI, [63]), a tool for measuring the severity of depression, may behave similarly. The BDI reflects an individual's current state of depression, meaning that it captures how a person is feeling at the specific time they take the test. Someone might score high on the BDI 1 day due to a stressful event, but their score could be much lower at a subsequent assessment if the stressor has been resolved. This interpretation may align with previous associations of PIT, mental health conditions and stress [64, 65, 66]. PIT as a dynamic state rather than a static trait has applications in the context of addiction recovery and aftercare, especially with respect to the effect of one's environment and the risk of recurrence of drug use. For example, adaptive approaches have been suggested to enable individuals in residential treatment settings to manage alcohol and drug cue exposure with personalized approaches and avoidance coping techniques [67]. If there are indeed fluctuations in an individual's response to environmental cues, they could serve as an indicator for potential relapse risk if meaningfully and timely measured. To track Pavlovian biases over time, it would be beneficial to develop a version of the PIT task that allows for repeated measurements.

### 4.2 fMRI Reliability

#### 4.2.1 Regional Reliability

The split‐half ICC values for the regional analysis indicate good split‐half reliability for both incongruent and congruent contrasts across both samples, with ICCs at or above 0.47 with many exceeding 0.60. This confirms the reliability of fMRI brain signals in the amygdala, VS, dmPFC and lPFC when processing congruent and incongruent outcomes in regions that have been previously associated with PIT and used to predict drinking trajectories [14].

However, test–retest reliability for the regional fMRI data, both the incongruent vs. congruent contrast (representing the interference PIT effect) and the motivational PIT effect contrast did not meet acceptable standards above ICC = 0.4. Despite this, certain regions (e.g., the VS in the clinical sample) demonstrated nonzero reliability within these contrasts, indicating at least some stability in these signals, albeit below acceptable thresholds. This result is somewhat comparable to 2015 findings from the monetary incentive delay task, where test–retest assessment over an interval greater than 2.5 years revealed significant temporal stability only in large incentive conditions, with intraclass correlations exceeding 0.50 for left nucleus accumbens activity during anticipation of large gains and right anterior insula activity during anticipation of large losses [68]. In both studies, variability in signal reliability, particularly under different conditions or contrasts, suggests that the long‐term stability of neural activity can be context dependent.

Regarding the interference PIT effect, this result is consistent with what is already evidenced more broadly in the literature. Bach and colleagues [43] demonstrated low mean test–retest reliability of a difference contrast and concluded that it resulted from the intercorrelation between the constituting task conditions, which eliminates parts of the shared variance and increases error variance. As the two constituent parts of the incongruent versus congruent contrast were highly correlated, eliminating their shared variance rendered the contrast almost ‘empty’—hence the low scores we observed. This phenomenon is also reflected in the results from Infantolino et al. [44], who noted very poor reliability of difference contrasts due to high correlations among task conditions. Other studies have also reached a similar conclusion [32, 56]. However, in our study, the constituent contrasts alone (congruent and incongruent) displayed fair‐to‐good reliability. When considering biomarker research, these contrasts may provide better utility to approximate the neural mechanisms associated with the PIT effect. An example of this contrast in use for this purpose can be found in [14]. Note, though, that some researchers warn that the excessive promotion of reliability at a study's onset could be detrimental to the validity of the study design and that one should proceed cautiously to ensure the integrity of the research [33].

The poor reliability of the motivational PIT effect (parametric) contrast may stem from several factors. In 2019, Schad and colleagues observed that the PIT effect of alcohol‐related cues was associated with increased functional activation in the nucleus accumbens, but only in abstainers. Therefore, one possibility is that motivationally relevant decisions interacted with the activation we observed and thus contributed to variability in the effect. In addition, changes in reward processing during early adulthood may have played a role in the instability of the motivational PIT contrast [69, 70]. Lastly, the neural basis of the motivational aspect of PIT may be more difficult to capture than our current approach allows.

#### 4.2.2 Global Reliability

We lastly assessed the global reliability of PIT. Using the Jaccard coefficients, we assessed the degree of overlap with each contrast/condition. Between both splits, the global neural responses for both the incongruent and congruent contrasts resulted in 47%–51% overlap in both samples, as indicated by the Jaccard coefficients. This once again reinforces the notion that whole‐brain activation patterns are comparable during PIT over short and extended periods. Consistent with the results about regional reliability, the split‐half and test–retest global reliability of the difference and motivational PIT effect contrasts showed poor reliability in both the clinical and developmental samples. Additionally, the test–retest results of the incongruent and congruent contrasts indicated between 29% and 35% overlap between test periods, regardless of sample. These findings overall indicate that while certain fMRI contrasts reflecting basic processes that are associated with PIT, particularly, the congruent and incongruent contrasts as well as the monetary trial onset, demonstrate strong and consistent stability (both longitudinally and cross‐sectionally), other contrasts are not as reliable. Therefore, there must be careful consideration in the application of these nonreliable contrasts/conditions in future fMRI PIT analyses.

From a developmental perspective, Koolschijn et al. [30] found that the test–retest reliability of a rule‐switch task varied with age. They observed fair to good reliability for adolescents and adults over a 3.5‐year interval but poor to fair reliability for children. They also noted that variability in neural activation patterns was significantly greater in children, suggesting developmental changes in brain regions related to performance monitoring. Our study's findings also relate to the recommendations and future directions outlined in Elliott *et al*. [32]. They discussed split‐half reliability (i.e., cross‐sectional) as a more practical measure for task‐based fMRI measures that are expected to change over time. If PIT is indeed more reflective of a dynamic state, then the internal consistency could be a more meaningful measure of reliability than test–retest measures. It is also worth considering that reliability, in general, highly depends on the model, as variations in modelling can result in different reliability outcomes (even with identical data). Employing a machine learning‐based approach to extract components and understand their associations with PIT may prove beneficial for future models [71, 72]. Additionally, task design improvements may also offer a way to enhance reliability. Future studies could assess whether variations in task mechanics or incentive structures influence the consistency of PIT neural activation patterns across time. For instance, a study examining the impact of gamification on the reliability of a Stroop task found that game elements, such as points and feedback, led to increased internal consistency and performance stability across sessions [73].

### 4.3 Limitations

Some limitations of this study should now be addressed. First, the studies from which these data originate were not designed to assess test–retest reliability, but rather to assess changes over time. A study designed specifically to assess reliability would have used a 24‐h interval, parallel versions and healthy participants. The 3‐year time difference between the baseline and follow‐up assessments is also substantial compared to other test–retest reliability studies. As we included a developmental cohort in our sample, many biological changes occurred between the baseline and follow‐up assessments. Further, those with alcohol dependence in the clinical sample were assessed directly after detoxification and another 3 weeks later. During this period, brain recovery occurs, which may interfere with our reliability analyses. Because of the data we used, we cannot separate error variance or variance due to change over time. Future studies using an adult, healthy sample could compare correlations between adjacent and nonadjacent time points to separate error variance from variance due to change over time. A decline in correlation across more distant measurements would suggest genuine change rather than error. By using two independent samples, we demonstrated robustness in the results; however, our results can only be generalized to our specific task design and may not be representative of other PIT tasks.

## 5 Conclusions

In summary, our study presents an analysis of the reliability of PIT effects, both behaviourally and through fMRI analysis. The behavioural aspect demonstrated excellent split‐half reliability in two independent samples, suggesting a strong degree of stability in the influence of Pavlovian cues on instrumental actions, despite the variability in test–retest reliability over different durations. This variability over time suggests that PIT may also reflect a dynamic state rather than simply capturing trait characteristics. Our analysis further revealed that specific brain regions, notably the VS, dmPFC and lPFC, exhibited reliable responses in the congruent and incongruent fMRI contrasts. These findings corroborate previous findings with respect to the role of these regions in PIT. However, the low reliability in the neural modulation of interference PIT difference and parametric modulation of the PIT effect contrast suggests that the neural basis of this aspect of PIT may be more difficult to capture than our current approach allows. The global reliability assessment (i.e., Jaccard coefficients) further confirmed the congruent and incongruent contrasts as reliable. Further studies are needed to compare statistical designs and contrasts to identify the most effective strategy for analyzing inter‐ and intra‐individual differences.

## Author Contributions

MJB drafted the manuscript and conducted the behavioural analysis. HC and JHF processed the fMRI data and conducted the fMRI analysis. MJB created the figure. MG, JHF, AH and MNS aided in the interpretation of the results. MNS and AH obtained funding and ethical approval for this study. All authors read, reviewed, and approved the final manuscript.

## Funding

This study was supported by the German Research Foundation (DFG: Deutsche Forschungsgemeinschaft Project Numbers 402170461 [TRR 265: Losing and Regaining Control over Drug Intake: Trajectories, Mechanisms, and Interventions], 186318919 [FOR 1617: Learning and Habitization as Predictors of the Development and Maintenance of Alcoholism], 454245598 [IRTG 2773: Risks and Pathomechanisms of Affective Disorders]) and 178833530 [SFB 940: Volition and Cognitive Control: Mechanisms, Modulators and Dysfunctions]).

## Supporting information


**Data S1:** Information regarding orthogonalization of the parametric regressors.
**Table S2:** A summary of the distribution of values obtained from the permutation tests of the behavioural analysis.
**Figure S3:** A series of histograms representing the distribution of values obtained from the permutation tests of the behavioural analysis.
**Table S4A:** A version of Table 3 including the Jaccard coefficients at the p < 0.05 and p < 0.001 thresholds.
**Table S4B:** An extended version of Table 3 outlining the regional and global reliability/stability of fMRI data including the confidence intervals.

## Data Availability

The data that support the findings of this study are openly available in NeuroVault at https://neurovault.org/collections/KJYQNYJO/, reference number https://identifiers.org/neurovault.collection:17843.
