# Supplementary material for: Behavioural and Neural Reliability of a Pavlovian‐to‐Instrumental Transfer Task
Source: Addict Biol. 2025 Dec 18;30(12):e70112. doi: 10.1111/adb.70112 (PMC12714411; doi:10.1111/adb.70112)
Supplement: Supplementary file 1 — Data S1: Information regarding orthogonalization of the parametric regressors. Table S2: A summary of the distribution of values obtained from the permutation tests of the behavioural analysis. Figure S3: A series of histograms representing the distribution of values obtained from the permutation tests of the behavioural analysis. Table S4A: A version of Table 3 including the Jaccard coefficients at the p < 0.05 and p < 0.001 thresholds. Table S4B: An extended version of Table 3 outlining the regional and global reliability/stability of fMRI data including the confidence intervals. [file ADB-30-e70112-s001.docx]

Supplementary Material

Behavioral and Neural Reliability of a Pavlovian-to-Instrumental Transfer Task

Matthew J. Belanger^1,2*^, Hao Chen^1*^, Juliane H. Fröhner^1^, Maria Garbusow^3,4^, Andreas Heinz^3^, Michael N. Smolka^1^

^1^Department of Psychiatry and Psychotherapy, Technische Universität Dresden, Dresden, Germany

^2^Department of Sociology, Social Policy, and Criminology, Faculty of Social Sciences, University of Stirling, Stirling, United Kingdom

^3^Department of Psychiatry and Neurosciences, Charité – Universitätsmedizin Berlin, Campus Charité Mitte, Berlin, Germany

^4^Department of Psychology, Clinical Psychology and Psychotherapy, MSB Medical School Berlin, Berlin, Germany.

*These authors contributed equally to this work.

Corresponding author:

Michael N. Smolka, MD
Section of Systems Neuroscience
Department of Psychiatry and Psychotherapy
Technische Universität Dresden
Würzburger Str. 35
01187 Dresden, Germany

Phone: +49 351 463 42201
Fax: +49 351 463 42202
E-Mail: [michael.smolka@tu-dresden.de](mailto:michael.smolka@tu-dresden.de)

Section S1: Information regarding orthogonalization of the parametric regressors.

The parametric modulators in this study were not orthogonalized with respect to the main regressors or to each other. When orthogonalization is enabled, the order of parametric modulators becomes important: the first modulator explains as much variance as possible; the second explains only the variance not accounted for by the first, and so forth. Because we have three modulators, this introduces arbitrariness into the model design. Additionally, in this setup, the interpretation of beta coefficients becomes more challenging, as they no longer reflect the independent contribution of each modulator. For these reasons, we turned off orthogonalization to avoid order dependency and to retain interpretability of the effects.

Table S2. A summary of the distribution of values obtained from the permutation tests of the behavioral analysis.

| PIT Effect | Sample | Time Point | Median | SD | Minimum | Q25 | Q75 | Maximum |
| --- | --- | --- | --- | --- | --- | --- | --- | --- |
| Interference | Dev | T1 | 0.92 | 0.01 | 0.87 | 0.92 | 0.93 | 0.96 |
|  |  | T2 | 0.95 | 0.01 | 0.91 | 0.94 | 0.95 | 0.98 |
|  | Clinical | T1 | 0.95 | 0.01 | 0.92 | 0.95 | 0.95 | 0.97 |
|  |  | T2 | 0.95 | 0.01 | 0.90 | 0.94 | 0.95 | 0.98 |
| Motivational | Dev | T1 | 0.94 | 0.01 | 0.90 | 0.94 | 0.95 | 0.97 |
|  |  | T2 | 0.96 | 0.01 | 0.94 | 0.96 | 0.97 | 0.98 |
|  | Clinical | T1 | 0.97 | 0.01 | 0.94 | 0.96 | 0.97 | 0.98 |
|  |  | T2 | 0.98 | 0.01 | 0.94 | 0.97 | 0.98 | 0.99 |

Figure S3. A series of histograms representing the distribution of values obtained from the permutation tests of the behavioral analysis.


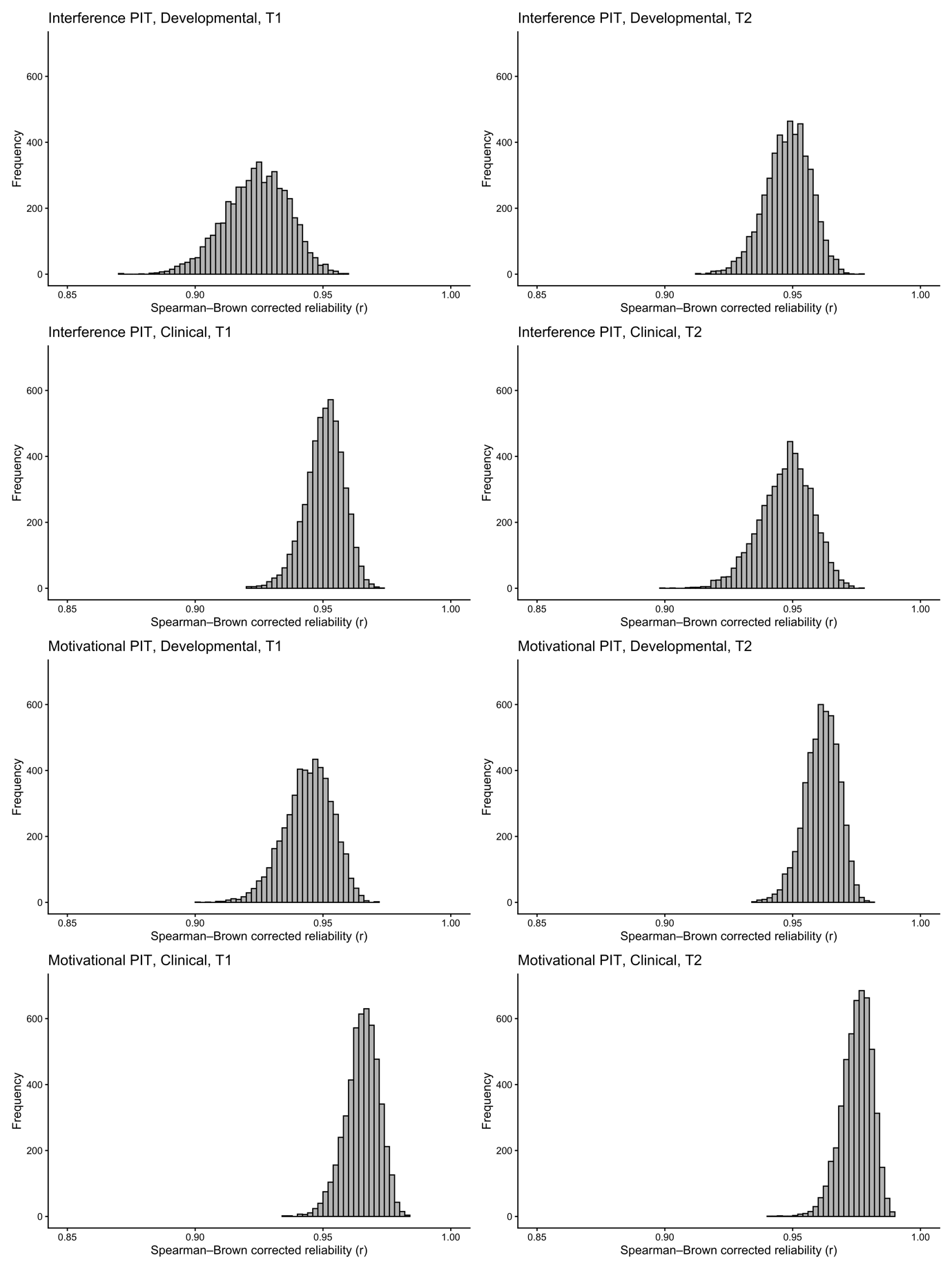


Table S4A. A version of Table 3 including the Jaccard coefficients at the p<.05 and p<.001 thresholds.

|  |  | **Incongruent** | | **Congruent** | | **Incongruent vs. congruent** | | **Monetary trial onset** | | **Motivation PIT effect (parametric)** | |
| --- | --- | --- | --- | --- | --- | --- | --- | --- | --- | --- | --- |
|  |  | **clinical** | **dev** | **clinical** | **dev** | **clinical** | **dev** | **clinical** | **dev** | **clinical** | **dev** |
| **Split-half T1** | **Amy** | 0.666 | 0.548 | 0.586 | 0.667 | 0.091 | -0.082 | 0.122 | 0.373 | -0.007 | -0.017 |
|  | **VS** | 0.639 | 0.468 | 0.671 | 0.624 | 0.086 | -0.002 | 0.295 | 0.443 | 0.071 | 0.027 |
|  | **lPFC** | 0.603 | 0.587 | 0.709 | 0.723 | 0.075 | 0.092 | 0.461 | 0.475 | -0.088 | 0.107 |
|  | **dmPFC** | 0.484 | 0.545 | 0.591 | 0.688 | 0.019 | 0.119 | 0.526 | 0.474 | -0.048 | 0.055 |
|  | ***Global p<.05*** | 0.493 | 0.475 | 0.493 | 0.491 | 0.014 | 0.017 | 0.306 | 0.329 | 0.020 | 0.012 |
|  | ***Global p<.01*** | 0.485 | 0.468 | 0.482 | 0.486 | 0.002 | 0.002 | 0.319 | 0.340 | 0.005 | 0.002 |
|  | ***Global p<.001*** | 0.497 | 0.484 | 0.491 | 0.504 | 0.000 | 0.000 | 0.317 | 0.368 | 0.000 | 0.001 |
| **Split-half T2** | **Amy** | 0.706 | 0.550 | 0.640 | 0.620 | -0.004 | 0.112 | 0.071 | 0.461 | 0.076 | -0.067 |
|  | **VS** | 0.701 | 0.582 | 0.607 | 0.587 | -0.007 | 0.001 | -0.060 | 0.333 | 0.034 | -0.065 |
|  | **lPFC** | 0.798 | 0.624 | 0.676 | 0.711 | 0.223 | 0.010 | 0.415 | 0.397 | 0.001 | -0.009 |
|  | **dmPFC** | 0.729 | 0.545 | 0.639 | 0.669 | 0.097 | -0.008 | 0.492 | 0.328 | -0.032 | -0.157 |
|  | ***Global p<.05*** | 0.515 | 0.491 | 0.487 | 0.493 | 0.018 | 0.017 | 0.284 | 0.327 | 0.018 | 0.018 |
|  | ***Global p<.01*** | 0.509 | 0.491 | 0.474 | 0.489 | 0.004 | 0.003 | 0.285 | 0.341 | 0.004 | 0.003 |
|  | ***Global p<.001*** | 0.521 | 0.515 | 0.483 | 0.511 | 0.000 | 0.000 | 0.282 | 0.364 | 0.000 | 0.001 |
| **Test-Retest** | **Amy** | 0.134 | 0.282 | 0.164 | 0.267 | 0.009 | -0.055 | 0.073 | 0.072 | 0.055 | -0.054 |
|  | **VS** | 0.285 | 0.330 | 0.302 | 0.253 | -0.051 | -0.136 | 0.198 | 0.116 | 0.170 | -0.035 |
|  | **lPFC** | 0.421 | 0.419 | 0.386 | 0.511 | -0.077 | -0.119 | 0.550 | 0.272 | -0.029 | 0.012 |
|  | **dmPFC** | 0.401 | 0.360 | 0.368 | 0.381 | -0.040 | -0.070 | 0.577 | 0.192 | -0.116 | 0.032 |
|  | ***Global p<.05*** | 0.364 | 0.370 | 0.324 | 0.356 | 0.010 | 0.018 | 0.264 | 0.273 | 0.016 | 0.014 |
|  | ***Global p<.01*** | 0.352 | 0.349 | 0.289 | 0.336 | 0.001 | 0.003 | 0.271 | 0.284 | 0.003 | 0.002 |
|  | ***Global p<.001*** | 0.355 | 0.344 | 0.273 | 0.330 | 0.001 | 0.000 | 0.280 | 0.303 | 0.000 | 0.000 |

The overlap results remain broadly comparable across thresholds (p<.001, p<.01, or p<.05). The Jaccard coefficient tends to increase with increasing overlapping voxel numbers. Still, it can decrease if the total number of activated voxels increases disproportionately. Therefore, lowering the threshold could simultaneously increase overlap and the total number of activated voxels. For specific contrasts with moderate overlap, such as the incongruent condition, adjusting the threshold in either direction (more liberal or more conservative) increased the Jaccard coefficient. According to these results, at the conservative threshold, the reduction in total activation did not compromise overlap to a remarkable degree, suggesting that the most strongly activated voxels were relatively consistent across sessions. At the liberal threshold, although the total number of activated voxels increased, the overlap also rose disproportionately, indicating that additional weaker, yet spatially consistent, activations contributed to the observed reliability. For contrasts with weaker neural responses (e.g., incongruent vs. congruent or motivational PIT), since the activation was generally low, lowering the threshold mainly increased the number of overlapping voxels, resulting in a higher Jaccard coefficient.

Table S4B. An extended version of Table 3 outlining the regional and global reliability/stability of fMRI data, including the confidence intervals.

| **Contrast** | **Sample** | **Reliability** | **ROI** | **ICC** | **CI_low** | **CI_high** |
| --- | --- | --- | --- | --- | --- | --- |
| incong | clinical | Splithalf_T1 | Amy | 0.666 | 0.57264 | 0.75934 |
| incong | clinical | Splithalf_T1 | VS | 0.639 | 0.53948 | 0.73809 |
| incong | clinical | Splithalf_T1 | lPFC | 0.603 | 0.49573 | 0.70943 |
| incong | clinical | Splithalf_T1 | dmPFC | 0.484 | 0.35537 | 0.61234 |
| incong | clinical | Splithalf_T2 | Amy | 0.706 | 0.62189 | 0.79016 |
| incong | clinical | Splithalf_T2 | VS | 0.701 | 0.61602 | 0.78654 |
| incong | clinical | Splithalf_T2 | lPFC | 0.798 | 0.73738 | 0.85911 |
| incong | clinical | Splithalf_T2 | dmPFC | 0.729 | 0.65022 | 0.8075 |
| incong | clinical | Test-Retest | Amy | 0.134 | -0.030306 | 0.75934 |
| incong | clinical | Test-Retest | VS | 0.285 | 0.13078 | 0.73809 |
| incong | clinical | Test-Retest | lPFC | 0.421 | 0.2829 | 0.70943 |
| incong | clinical | Test-Retest | dmPFC | 0.401 | 0.26067 | 0.61234 |
| incong | dev | Splithalf_T1 | Amy | 0.548 | 0.43007 | 0.66501 |
| incong | dev | Splithalf_T1 | VS | 0.468 | 0.33741 | 0.59933 |
| incong | dev | Splithalf_T1 | lPFC | 0.587 | 0.47745 | 0.69723 |
| incong | dev | Splithalf_T1 | dmPFC | 0.545 | 0.42722 | 0.66304 |
| incong | dev | Splithalf_T2 | Amy | 0.550 | 0.43261 | 0.66675 |
| incong | dev | Splithalf_T2 | VS | 0.582 | 0.47127 | 0.69307 |
| incong | dev | Splithalf_T2 | lPFC | 0.624 | 0.52117 | 0.72618 |
| incong | dev | Splithalf_T2 | dmPFC | 0.545 | 0.42681 | 0.66276 |
| incong | dev | Test-Retest | Amy | 0.282 | 0.12781 | 0.43661 |
| incong | dev | Test-Retest | VS | 0.330 | 0.17998 | 0.47907 |
| incong | dev | Test-Retest | lPFC | 0.419 | 0.28018 | 0.55692 |
| incong | dev | Test-Retest | dmPFC | 0.360 | 0.21368 | 0.50578 |
| cong | clinical | Splithalf_T1 | Amy | 0.586 | 0.47552 | 0.69593 |
| cong | clinical | Splithalf_T1 | VS | 0.671 | 0.57848 | 0.76304 |
| cong | clinical | Splithalf_T1 | lPFC | 0.709 | 0.62555 | 0.79242 |
| cong | clinical | Splithalf_T1 | dmPFC | 0.591 | 0.4816 | 0.70001 |
| cong | clinical | Splithalf_T2 | Amy | 0.640 | 0.54119 | 0.7392 |
| cong | clinical | Splithalf_T2 | VS | 0.607 | 0.50112 | 0.71299 |
| cong | clinical | Splithalf_T2 | lPFC | 0.676 | 0.58524 | 0.76731 |
| cong | clinical | Splithalf_T2 | dmPFC | 0.639 | 0.54021 | 0.73857 |
| cong | clinical | Test-Retest | Amy | 0.164 | 0.00049614 | 0.32702 |
| cong | clinical | Test-Retest | VS | 0.302 | 0.1493 | 0.45427 |
| cong | clinical | Test-Retest | lPFC | 0.386 | 0.24317 | 0.52872 |
| cong | clinical | Test-Retest | dmPFC | 0.368 | 0.22342 | 0.51341 |
| cong | dev | Splithalf_T1 | Amy | 0.667 | 0.57346 | 0.75986 |
| cong | dev | Splithalf_T1 | VS | 0.624 | 0.52168 | 0.72651 |
| cong | dev | Splithalf_T1 | lPFC | 0.723 | 0.6428 | 0.80298 |
| cong | dev | Splithalf_T1 | dmPFC | 0.688 | 0.59977 | 0.77643 |
| cong | dev | Splithalf_T2 | Amy | 0.620 | 0.51611 | 0.72287 |
| cong | dev | Splithalf_T2 | VS | 0.587 | 0.47678 | 0.69678 |
| cong | dev | Splithalf_T2 | lPFC | 0.711 | 0.62855 | 0.79427 |
| cong | dev | Splithalf_T2 | dmPFC | 0.669 | 0.57669 | 0.76191 |
| cong | dev | Test-Retest | Amy | 0.267 | 0.11093 | 0.42258 |
| cong | dev | Test-Retest | VS | 0.253 | 0.095507 | 0.40963 |
| cong | dev | Test-Retest | lPFC | 0.511 | 0.38685 | 0.63482 |
| cong | dev | Test-Retest | dmPFC | 0.381 | 0.23795 | 0.52468 |
| difference | clinical | Splithalf_T1 | Amy | 0.091 | -0.074914 | 0.2578 |
| difference | clinical | Splithalf_T1 | VS | 0.086 | -0.080688 | 0.25236 |
| difference | clinical | Splithalf_T1 | lPFC | 0.075 | -0.09156 | 0.24206 |
| difference | clinical | Splithalf_T1 | dmPFC | 0.019 | -0.1486 | 0.1868 |
| difference | clinical | Splithalf_T2 | Amy | -0.004 | -0.17193 | 0.16358 |
| difference | clinical | Splithalf_T2 | VS | -0.007 | -0.17494 | 0.16056 |
| difference | clinical | Splithalf_T2 | lPFC | 0.223 | 0.063039 | 0.38195 |
| difference | clinical | Splithalf_T2 | dmPFC | 0.097 | -0.068788 | 0.26355 |
| difference | clinical | Test-Retest | Amy | 0.009 | -0.15846 | 0.17703 |
| difference | clinical | Test-Retest | VS | -0.051 | -0.21835 | 0.11629 |
| difference | clinical | Test-Retest | lPFC | -0.077 | -0.24326 | 0.0903 |
| difference | clinical | Test-Retest | dmPFC | -0.040 | -0.20767 | 0.1273 |
| difference | dev | Splithalf_T1 | Amy | -0.082 | -0.24894 | 0.08431 |
| difference | dev | Splithalf_T1 | VS | -0.002 | -0.16947 | 0.16605 |
| difference | dev | Splithalf_T1 | lPFC | 0.092 | -0.074789 | 0.25792 |
| difference | dev | Splithalf_T1 | dmPFC | 0.119 | -0.046545 | 0.28424 |
| difference | dev | Splithalf_T2 | Amy | 0.112 | -0.053894 | 0.27744 |
| difference | dev | Splithalf_T2 | VS | 0.001 | -0.1666 | 0.16892 |
| difference | dev | Splithalf_T2 | lPFC | 0.010 | -0.1576 | 0.17788 |
| difference | dev | Splithalf_T2 | dmPFC | -0.008 | -0.17535 | 0.16015 |
| difference | dev | Test-Retest | Amy | -0.055 | -0.22197 | 0.11255 |
| difference | dev | Test-Retest | VS | -0.136 | -0.30056 | 0.028764 |
| difference | dev | Test-Retest | lPFC | -0.119 | -0.28417 | 0.046615 |
| difference | dev | Test-Retest | dmPFC | -0.070 | -0.23675 | 0.09714 |
| monetary | clinical | Splithalf_T1 | Amy | 0.122 | -0.043149 | 0.28737 |
| monetary | clinical | Splithalf_T1 | VS | 0.295 | 0.14207 | 0.44835 |
| monetary | clinical | Splithalf_T1 | lPFC | 0.461 | 0.32868 | 0.59295 |
| monetary | clinical | Splithalf_T1 | dmPFC | 0.526 | 0.40494 | 0.64755 |
| monetary | clinical | Splithalf_T2 | Amy | 0.071 | -0.12687 | 0.2081 |
| monetary | clinical | Splithalf_T2 | VS | -0.060 | -0.22699 | 0.10733 |
| monetary | clinical | Splithalf_T2 | lPFC | 0.415 | 0.27651 | 0.55415 |
| monetary | clinical | Splithalf_T2 | dmPFC | 0.492 | 0.36518 | 0.61939 |
| monetary | clinical | Test-Retest | Amy | 0.073 | -0.094032 | 0.23971 |
| monetary | clinical | Test-Retest | VS | 0.198 | 0.036623 | 0.35901 |
| monetary | clinical | Test-Retest | lPFC | 0.550 | 0.43328 | 0.66722 |
| monetary | clinical | Test-Retest | dmPFC | 0.577 | 0.46515 | 0.68894 |
| monetary | dev | Splithalf_T1 | Amy | 0.373 | 0.22903 | 0.51777 |
| monetary | dev | Splithalf_T1 | VS | 0.443 | 0.30865 | 0.5782 |
| monetary | dev | Splithalf_T1 | lPFC | 0.475 | 0.34498 | 0.60483 |
| monetary | dev | Splithalf_T1 | dmPFC | 0.474 | 0.34422 | 0.60428 |
| monetary | dev | Splithalf_T2 | Amy | 0.461 | 0.32929 | 0.5934 |
| monetary | dev | Splithalf_T2 | VS | 0.333 | 0.18421 | 0.48245 |
| monetary | dev | Splithalf_T2 | lPFC | 0.397 | 0.25608 | 0.53863 |
| monetary | dev | Splithalf_T2 | dmPFC | 0.328 | 0.17781 | 0.47733 |
| monetary | dev | Test-Retest | Amy | 0.072 | -0.09458 | 0.23919 |
| monetary | dev | Test-Retest | VS | 0.116 | -0.049312 | 0.28168 |
| monetary | dev | Test-Retest | lPFC | 0.272 | 0.11662 | 0.42733 |
| monetary | dev | Test-Retest | dmPFC | 0.192 | 0.030353 | 0.35351 |
| motivation | clinical | Splithalf_T1 | Amy | -0.007 | -0.17486 | 0.16064 |
| motivation | clinical | Splithalf_T1 | VS | 0.071 | -0.095663 | 0.23816 |
| motivation | clinical | Splithalf_T1 | lPFC | -0.088 | -0.25481 | 0.078087 |
| motivation | clinical | Splithalf_T1 | dmPFC | -0.048 | -0.21568 | 0.11906 |
| motivation | clinical | Splithalf_T2 | Amy | 0.076 | -0.091048 | 0.24255 |
| motivation | clinical | Splithalf_T2 | VS | 0.034 | -0.13328 | 0.20185 |
| motivation | clinical | Splithalf_T2 | lPFC | 0.001 | -0.16661 | 0.16891 |
| motivation | clinical | Splithalf_T2 | dmPFC | -0.032 | -0.19965 | 0.13552 |
| motivation | clinical | Test-Retest | Amy | 0.055 | -0.11227 | 0.22224 |
| motivation | clinical | Test-Retest | VS | 0.170 | 0.0074834 | 0.33327 |
| motivation | clinical | Test-Retest | lPFC | -0.029 | -0.1966 | 0.13864 |
| motivation | clinical | Test-Retest | dmPFC | -0.116 | -0.28102 | 0.050023 |
| motivation | dev | Splithalf_T1 | Amy | -0.017 | -0.18488 | 0.15054 |
| motivation | dev | Splithalf_T1 | VS | 0.027 | -0.14114 | 0.19414 |
| motivation | dev | Splithalf_T1 | lPFC | 0.107 | -0.059045 | 0.27265 |
| motivation | dev | Splithalf_T1 | dmPFC | 0.055 | -0.11212 | 0.22238 |
| motivation | dev | Splithalf_T2 | Amy | -0.067 | -0.23439 | 0.099603 |
| motivation | dev | Splithalf_T2 | VS | -0.065 | -0.23239 | 0.1017 |
| motivation | dev | Splithalf_T2 | lPFC | -0.009 | -0.17698 | 0.15852 |
| motivation | dev | Splithalf_T2 | dmPFC | -0.157 | -0.32053 | 0.0067357 |
| motivation | dev | Test-Retest | Amy | -0.054 | -0.22113 | 0.11342 |
| motivation | dev | Test-Retest | VS | -0.035 | -0.20252 | 0.13259 |
| motivation | dev | Test-Retest | lPFC | 0.012 | -0.15588 | 0.17959 |
| motivation | dev | Test-Retest | dmPFC | 0.032 | -0.13569 | 0.19949 |
